# First person – Alicia Estacio-Gómez

**DOI:** 10.1242/bio.053264

**Published:** 2020-06-03

## Abstract

First Person is a series of interviews with the first authors of a selection of papers published in Biology Open, helping early-career researchers promote themselves alongside their papers. Alicia Estacio-Gómez is first author on ‘Dynamic neurotransmitter specific transcription factor expression profiles during *Drosophila* development’, published in BiO. Alicia is a research associate (postdoc) in the lab of Tony D. Southall at the Department of Life Sciences, Imperial College London, London, UK, investigating neural specification during development and gene regulation.


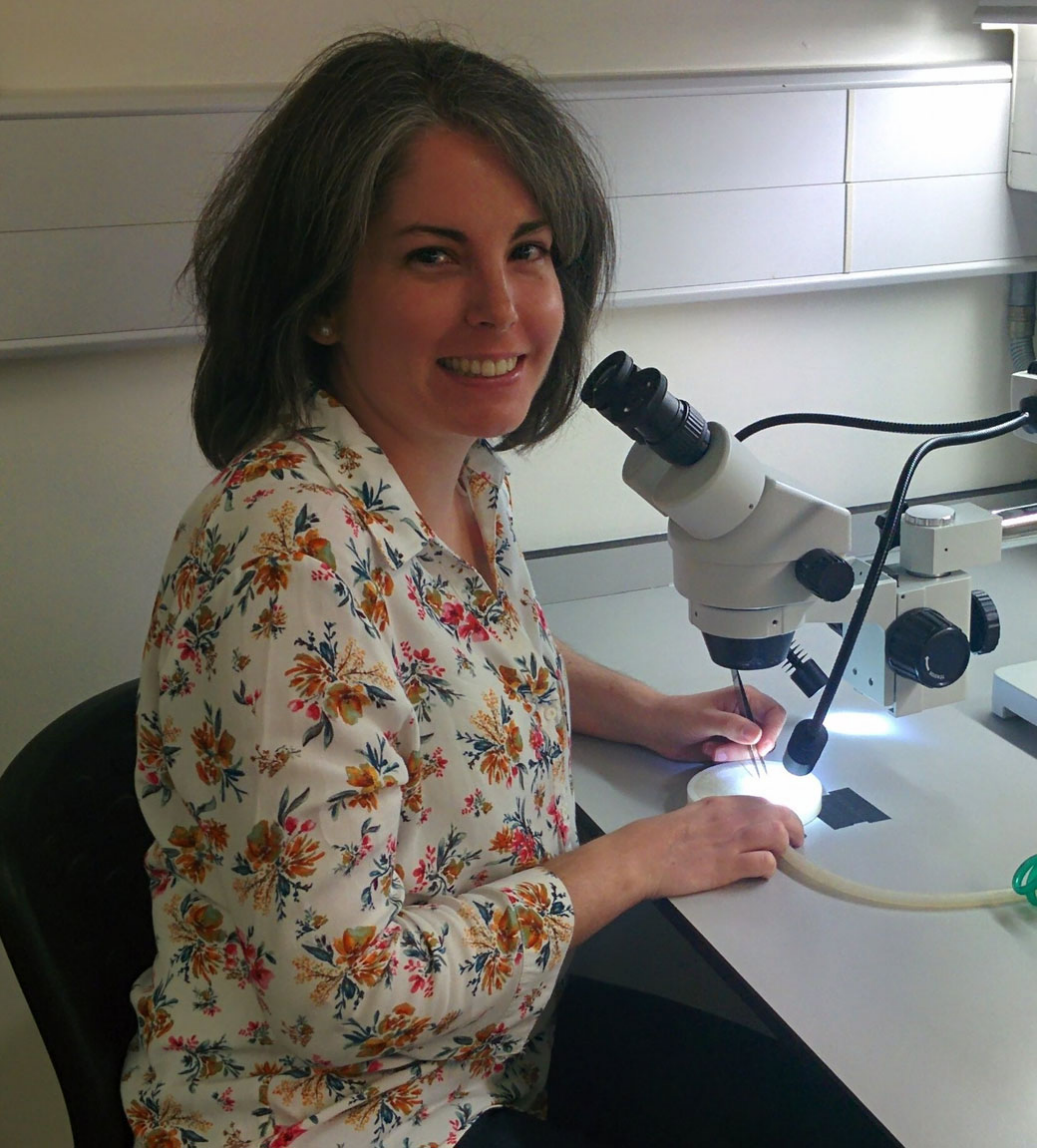


**Alicia Estacio-Gómez**

**What is your scientific background and the general focus of your lab?**

I studied Biology at the Universidad Autónoma de Madrid, Spain. Then, I stayed to obtain a PhD in Molecular Biology. I joined the laboratory of Dr Fernando J. Díaz-Benjumea, in Centro Biología Molecular Severo Ochoa, also in Madrid, where I studied the mechanisms of neuronal specification of a small set of neurons located in the central nervous system of *Drosophila*. After that, I moved to London to start my postdoc in the lab of Dr Tony D. Southall, at Imperial College London, UK, where I have investigated how neurons acquired their neurotransmitter identity. The general focus of the lab is to understand the gene regulatory mechanisms behind neuronal differentiation.

**How would you explain the main findings of your paper to non-scientific family and friends?**

The human nervous system is one of the most complex tissues known to mankind, it contains 86 billion neurons. Even more importantly, it possesses a great set of diverse cell types, able to communicate harmoniously, and react to external stimuli.

One long-standing question in developmental neurobiology is how this cellular diversity is achieved throughout development, since all neural cells arise from a limited and homogenous number of progenitor cells. How do they become unique? In principle, the genes expressed by a neuron can be used as a proxy to identify their function. Here, I have investigated what genes are differentially expressed in three neuronal populations of the central nervous system of *Drosophila*, with the initial hypothesis of whether a gene battery could predict a specific neural identity. My data suggests that there does not seem to be a generic code, and neurons are able to acquire the same neurotransmitter identity by using different genes.

**What are the potential implications of these results for your field of research?**

Even though the neurotransmitter identity of a neuron dictates its function, it is a trait that has not been systematically studied so far, mainly due to the lack of tools. Here we provide a comprehensive study where we identify genes that are differentially expressed in cholinergic, GABAergic, and glutamatergic neurons at three developmental points. We have uncovered new roles for genes not previously described to be relevant in neural specification, and we have also confirmed the importance of the genes *acj6* and *Ets65A* in the establishment of cholinergic identity, through its binding to the DNA regulatory elements of a key gene involved in making cholinergic neurons. Considering the strong conservation of neurotransmitter specification mechanisms, our data constitutes a useful resource for both *Drosophila* and other animal models researchers.

**What has surprised you the most while conducting your research?**

I was surprised to discover how complex and elegantly wired the brain of a humble fruit fly can be, but yet, how the main principles seem to be conserved throughout evolution. On the practical side, I was a bit taken aback to realise that, despite all the technology improvements, there are not that many great antibodies against some of the neurotransmitters, or at least, not that have worked in my hands!

“…there are not that many great antibodies against some of the neurotransmitters, or at least, not that have worked in my hands!”

**What, in your opinion, are some of the greatest achievements in your field and how has this influenced your research?**

I think that our technical ability has improved enormously in the last few decades. You can have access to virtually any fly genotype you want; if the fly community has not generated it yet, you can do it yourself by different means: CRISPR/Cas9, swapping of different cassettes within MiMIC lines, ‘homemade’ cloning, company cloning, etc. The possibilities are endless. What's more, high throughput techniques such as next-generation, and single-cell RNA sequencing are routinely used in many labs. What this means to the field of neurobiology is that we are beginning to decipher the cellular physiology of individual neurons, and with that comes the understanding of how specific neuronal circuits are established.

**Figure:**
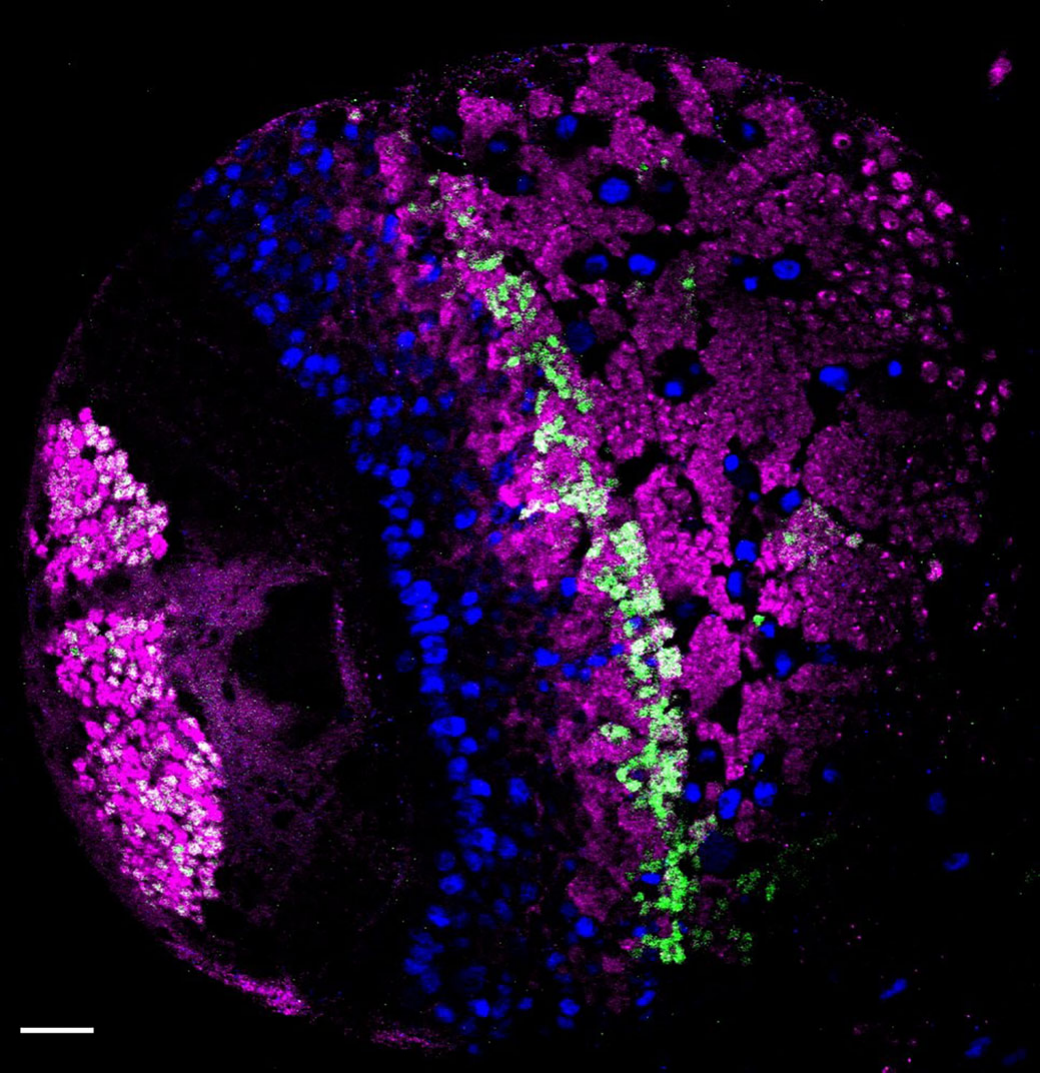
**Left brain lobe of a third instar larvae.** Ets65A is labelled with GFP (green), neural stem cells are visible with anti-deadpan, in blue, and neurons with anti-elav, in magenta. Our data suggested that the long isoform of *Ets65A* could act as a repressor of cholinergic fate. Scale bar: 20 µm.

**What changes do you think could improve the professional lives of early-career scientists?**

Job security could do with some improvement. It is a bit challenging to settle down, and advance in your personal life when most of the contracts offered to postdocs are on a fixed-term basis.

Also, a healthy work-life balance should be a priority, and Universities and research institutes could provide on-site childcare facilities, longer shared parental leaves, etc.

**What's next for you?**

I would like to continue working on research using fruit flies, and follow up on some of the findings I presented here, but also to deepen my knowledge in the characterization of novel regulatory mechanisms of transcription. On the personal side, I have just had baby twins, so finding the perfect balance between the lab, and family is definitely going to keep me busy for the next couple of years!
